# A UK-Wide Study Employing Natural Language Processing to Determine What Matters to People about Brain Health to Improve Drug Development: The Electronic Person-Specific Outcome Measure (ePSOM) Programme

**DOI:** 10.14283/jpad.2021.30

**Published:** 2021-06-09

**Authors:** Stina Saunders, G. Muniz-Terrera, S. Sheehan, C.W. Ritchie, S. Luz

**Affiliations:** 1 Centre for Clinical Brain Sciences, University of Edinburgh, Edinburgh, UK; 2 Usher Institute of Population Health Sciences and Informatics; Molecular, Genetic and Population Health Sciences, University of Edinburgh, Edinburgh, UK; 3 Brain Health Scotland, Scotland, UK

**Keywords:** Clinically meaningful change, electronic patient reported outcome measures, Alzheimer's disease, outcome measures, brain health

## Abstract

**Background:**

It is important to use outcome measures for novel interventions in Alzheimer's disease (AD) that capture the research participants' views of effectiveness. The electronic Person-Specific Outcome Measure (ePSOM) development programme is underpinned by the need to identify and detect change in early disease manifestations and the possibilities of incorporating artificial intelligence in outcome measures.

**Objectives:**

The aim of the ePSOM programme is to better understand what outcomes matter to patients in the AD population with a focus on those at the pre-dementia stages of disease. Ultimately, we aim to develop an app with robust psychometric properties to be used as a patient reported outcome measure in AD clinical trials.

**Design:**

We designed and ran a nationwide study (Aug 2019 - Nov 2019, UK), collecting primarily free text responses in five pre-defined domains. We collected self-reported clinical details and sociodemographic data to analyse responses by key variables whilst keeping the survey short (around 15 minutes). We used clustering and Natural Language Processing techniques to identify themes which matter most to individuals when developing new treatments for AD.

**Results:**

The study was completed by 5,808 respondents, yielding over 80,000 free text answers. The analysis resulted in 184 themes of importance. An analysis focusing on key demographics to explore how priorities differed by age, gender and education revealed that there are significant differences in what groups consider important about their brain health.

**Discussion:**

The ePSOM data has generated evidence on what matters to people when developing new treatments for AD that target secondary prevention and therein maintenance of brain health. These results will form the basis for an electronic outcome measure to be used in AD clinical research and clinical practice.

## Introduction

**A**ttempts to develop disease modifying therapies for Alzheimer's disease (AD) started over 20 years ago with little success to date. A recent estimate of the costs of AD was US$818B, which is equivalent to the combined GDP of Indonesia, The Netherlands, and Turkey (1).

The lack of progress in finding a pharmacological treatment for AD is however at odds with a rapid development in the understanding of the pathology of AD suggesting that clinical trial design and delivery may partially account for a lack of progress with insensitive outcome measures lacking clinical meaningfulness also playing a part in this lack of progress. It has been shown that the disease process starts long before an individual becomes symptomatic or eventually, the dementia syndrome manifests (2, 3). Increasingly, we are exploring AD processes at earlier disease stages through examining at-risk populations in mid-life which helps identify the earliest manifestation of declining brain health. In the absence of pharmacological interventions, it is estimated that approximately 40% of dementia cases could be prevented by targeting epidemiologically derived modifiable risk factors (4). Changes occurring years earlier than dementia develops have been observed in at-risk populations using exploratory and sensitive computerised tests assessing e.g. allocentric and egocentric spatial processing (5). These test results correlate with brain imaging findings in hippocampal subfields known to be sensitive to amyloid derived neurotoxicity (6); as well as in changes to brain β-amyloid in at risk populations aged between 63–81 years old who did not have dementia (7).

Whilst there are global initiatives focusing on dementia prevention through risk factor modification (8, 9), there remains a major and complementary need for effective AD pharmacological interventions. Irrespective of the type of intervention to reduce incident dementia rates, the fact is that these studies will engage at risk populations who will be, to the most part, in mid-life and healthy. Currently, there are 31 AD drugs being tested in Phase III clinical trials (19 of which are disease modifying) (10). We argue that using outcome measures assessing clinical symptoms and functioning in earlier disease stages is less valid than biological measures of disease and what the individual considers personally meaningful from a treatment. A treatment's success should therefore be determined not only by the impact on the individual's disease (as evidenced by biomarker change) but also by its effect on related well-being (as measured by patient reported outcomes).

To this end, whilst it is currently proposed by regulators that AD trials measure cognition as the primary outcome, as trials move to an earlier disease stage it could be argued that many commonly used (cognitive) measures lack ecological validity and are not sensitive enough to detect changes in the earlier stages of the AD continuum where the ideal intervention should take place (11). Moreover, it is recommended by both the US Food and Drug Administration (FDA) (12) and European Medicines Agency (EMA) (13) that AD trials incorporate measures which capture clinically meaningful results to the individual. Patient reported outcome measures (PROMs) are developed for the incorporation of the person's own perspective regarding their treatment, though these measures are currently not used in AD clinical trials (14). PROMs reflect an individual's view on what they define as an effective treatment and consider a meaningful change. Notably, PROMs are already more widely used in other disease areas. For example, a recent study of nearly 100,000 clinical trials published on clinicaltrials.gov found that a PROM had been used in 27% of all trials, primarily in oncology (15).

In light of the drive towards early detection, looking at younger at-risk populations and the main regulators' recommendation for clinically meaningful outcome measures, we have established the electronic Person Specific Outcome Measure (ePSOM) development programme. As the target population in dementia prevention research is an at-risk population, our group took the view that what matters to people when developing new treatments for AD is approached by way of maintenance of brain health (16, 17). The ePSOM programme consists of four sequential steps, ultimately aiming to employ new technology to create an outcome measure to be used in AD clinical research and practice. This will be in the form of an outcome app used on any screen-based device which will assess aspects specific to the individual using it. At the start of the programme, we reviewed literature around PROMs in AD clinical trials which informed our focus group study with people with memory concerns, healthy volunteers and health care professionals (18). The focus group study yielded five domains of importance for what matters to people about brain health. These domains formed the basis for the next stage of the ePSOM development programme. In this paper, we report on a large UK-wide population-based study to understand what matters to people when developing new treatments for Alzheimer's disease. We consider the respondents to the ePSOM study a representative population of individuals who may be enrolled in dementia primary and secondary prevention clinical trials and characterise what matters to people about brain health focusing on key demographic groups.

## Methods

We designed and ran a UK-wide population-based online study collecting primarily free text answers (see Appendix 1). The study built on a previously run focus group study which yielded five domains of importance about brain health. The study obtained ethics approval from the ACCORD Medical Research Ethics Committee in Edinburgh, Scotland. The ePSOM study ran from Aug 2019 – Dec 2019 and was divided into sections, starting with an introductory video and informed consent.

**Figure 1:**
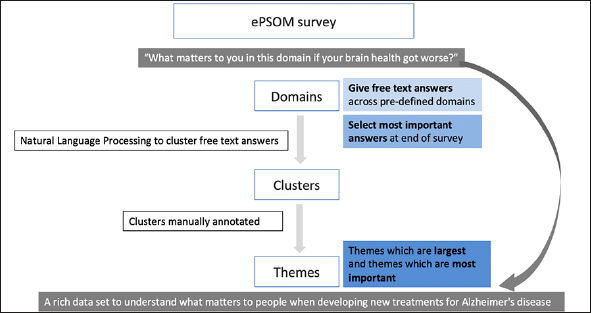
Natural Language Processing techniques used to analyse the survey data

The study was open to anyone over the age of 18 and was launched primarily via Alzheimer's Research UK media channels through e-mails to individuals registered on their mailing lists and a social media campaign (with social media support from other dementia related organisations). We collected key sociodemographic and clinical data such as having been seen by a doctor because of any brain health issues though the primary method of the survey used a qualitative approach. Respondents were presented with the five domains derived from the earlier focus group work to orientate and channel free text responses: [1] Everyday functioning; [2] Sense of Identity; [3] Relationships and Social Connections; [4] Enjoyable Activities and [5] Thinking problems. They were then asked to provide free text answers on what they would like to retain or keep being able to do in those domains if their brain health got worse. At the end of the study, respondents were asked to identify five answers across all the answers they had given which they consider the most important. We used Natural Language Processing (NLP) techniques to analyse the free text data (see Figure 1).

Free text answers were collected across five pre-defined domains. These answers were clustered, leading to specific themes of what matters to people about brain health

### Step 1: Natural Language Processing to create clusters

We used NLP to create clusters of semantically similar free text answers. These clusters were then manually annotated with appropriate labels. We refer to the finally labelled clusters derived from this stepped NLP-manual annotation process as “themes”.

NLP employed word embeddings trained on vast amounts of text data to achieve fine-grained representation of semantic regularities in text. We were thus able to build robust representations of free text answers. To begin, “stop words” (i.e. words that occur very frequently and contribute little to semantic content) and punctuation were removed from the free text answers. The resulting texts were then converted to numerical vector representations by using GloVe vectors (19) to generate sentence embeddings. These vectors encode semantic relationships between words and can be used to cluster semantically similar text segments. This allowed us to use automated methods to identify words, and thus answers, of a similar “theme” or meaning. The K-means clustering algorithm was used to cluster the answer embeddings within each of the five domains. The K parameter, that is, the desired number of automatic clusters per domain, was determined analytically. The goal was to generate fine grained clusters which contain semantically similar answers while avoiding overfitting or creating so many clusters that important themes are not revealed. We found when the number of important items in the largest cluster changes by less than 10, between each of the previous five increments of K, that the majority of the clusters also exhibited minor changes in the number of important items. Using this criterion, we chose a value of 151 clusters across all five domains. This method resulted in a total of 755 clusters of free text answers, or 151 clusters for each of the five domains.

### Step 2: Manual Annotation to create themes

The clusters that emerged within each of the five domains were reordered so that semantically similar clusters appeared close together. This was achieved using hierarchical clustering on the cluster centroids. We used the reordered clusters for manual annotation in each of the five domains. Each cluster was represented by the 200 most frequent unique answers, after punctuation and stopwords were removed. The annotation goals were to combine any clusters which fit together, exclude uninterpretable clusters and label the final clusters thus deriving the final themes. Six authors of the current paper annotated two domains each, ensuring two separate people analysed a single domain, which helped ensure inter-rater reliability between domains. Finally, two of the authors did quality control across the five domains and homogenised the labels across domains.

### Statistical analyses

In this paper, we focus our analyses on key demographic groups: age (up to the age of 64 / age 65 and older); gender (men / women) and education (no degree / degree and higher). We present the largest themes for each of these demographic groups as well as themes which were identified as particularly important most often in the final question on the study forms. For both of these analyses, we report percentages for each theme by key demographic groups. As the demographic groups are unbalanced in terms of the number of respondents we use percentages rather than the absolute number of answers in the statistical analyses. The percentages are derived by dividing the count of answers within the demographic group by the total number of answers in that demographic group, thus providing proportions for comparison when dealing with imbalanced demographics. It should be noted that respondents were not bounded by a minimum or maximum number of free text answers they could give in each domain.

Finally, we conducted a Chi-squared test to analyse whether the differences in percentages between demographic groupings' answers within each theme were statistically significant. A p-value of <0.01 was used in statistical significance testing.

## Results

The study was completed by 5,808 people from across the UK. They provided a total of 82,514 free text answers. These were clustered using automated NLP techniques resulting in 151 clusters in each of the five pre-defined domains, a total of 755 automated clusters across all domains, as described. Subsequent analysis reduced the number of clusters to 334 (due to a cluster being represented in two or more domains) which were all manually annotated by the research team. Many of the same themes emerged from different domains (e.g., the theme of Walking in the “Enjoyable activities” domain as well as the “Everyday activities” domain). After merging themes with the same label in different domains, the final number of unique themes was 184. Some respondents used more generic language (e.g., “Maintaining independence”) whereas others were more specific (e.g., “Driving”). Using NLP methods for free text analysis means that, in this example, the “Maintaining independence” theme contains 1100 answers, most containing either the word “independent” or “independence”. Analytically, this is therefore not a general theme for answers which relate to the concept of independence, but a cluster of answers in which the respondents are directly referencing the word independence as something which is important for them to maintain. This has therefore resulted in themes which are more or less specific but directly reflect the language used by the respondents.

### Pre-defined answers: Characteristics of the ePSOM survey sample

The characteristics of the 5,808 respondents are presented above (see Table 1).

**Table 1:** ePSOM survey respondent characteristics

ePSOM survey characteristics		
N	5808	
Male	1331 (23.1 %)	
Female	4463 (76.9%)	
Age	Mean: 58.6 (SD=13.8)	
Work status	Retired	2105 (36.2%)
	Working	1537 (26.5%)
	Part time paid work	767 (13.2%)
	Self-employed	397 (6.8%)
	Other (includes volunteering)	1002 (17.2%)
Education	Degree	2303 (39.7%)
	Postgraduate degree	1380 (23.8%)
	High school	971 (16.7%)
Marital status	Married	3684 (63.4%)
	Single	969 (16.7%)
	Divorced	630 (10.8%)
Ever supported a relative, friend or neighbour with dementia?	Yes	4281 (73.7%)
	No	1453 (25.0%)
Anyone assist in completing the survey?	No	5734 (98.7%)
	Yes	74 (0.03%)

### Free text answers: An analysis of important themes by key demographics

**Figure 2:**
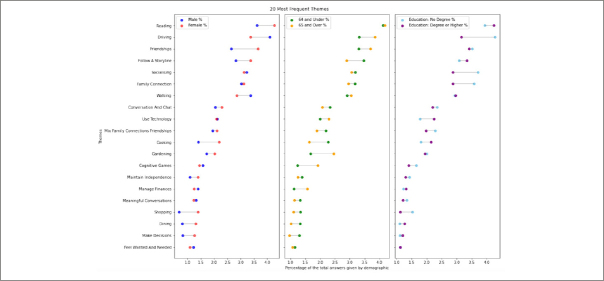
What matters to people about brain health? The survey received 82,514 free text answers which were clustered into 184 themes

We used NLP techniques and manual annotation to group individual free text answers into clusters and then themes respectively. The most frequent themes across all demographics were reading, driving, friendships and following a storyline (Figure 2). We also calculated the proportion of answers within each key demographic, expressed as percentages of the total answers given by that demographic group.

This figure shows themes which were mentioned the most, broken down by key demographics. Full figure of the survey themes in Appendix 2.

**Figure 3:**
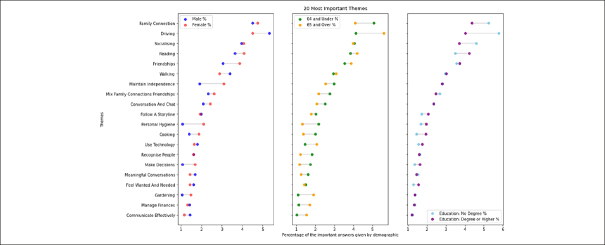
What matters to people about brain health?

At the end of the survey, respondents were asked to identify the five most important answers to them across all their answers. We used this metric to rank the themes in terms of being selected as particularly important and observed a subtle difference between the largest themes (themes mentioned the most frequently) and themes which are identified as the most important. The 5 top important themes across all demographics were family connections, driving, socializing, reading and friendships (Figure 3).

This figure shows themes with the highest number of answers selected as particularly important by key demographics. Full figure of themes with the most important answers in Appendix 3.

### Cross-Tabulations of key demographics

The following tables show statistically significant proportional differences in theme sizes (Table 2) and identifying themes as particularly important (Table 3), focusing on demographic group dyads (younger vs older; men vs women; individuals with no degree vs individuals with a degree or higher).

**Table 2:** Top 10 themes selected as particularly important which had the highest Chi square values representing greater differentiation between demographic groups by age (younger/older); gender (male/female) and education (no degree / degree and higher)

Theme	Age 64 and under	Age 65 and older	Men	Women	Education: no degree	Education: degree or higher	Chi-square
Working	√						72
Cognitive Games		√					38
Gardening		√					31
Driving		√					30
Golf		√					30
Mix Board Games and Cards		√					29
Personal Hygiene	√						25
Pets	√						21
Walk Dogs	√						16
Running	√						15
Golf			√				73
Sport			√				44
Fishing			√				32
Needlework				√			30
Cycling			√				28
Personal Hygiene				√			27
Maintain Independence				√			23
Water Sports			√				18
Concentrate and Understand Books			√				18
Music			√				17
Driving					√		42
Walk Dogs					√		14
Socialising					√		12
Traveling						√	11
Shopping					√		11
Going on Holidays					√		10
Remember Love					√		10
Family Connection					√		10
Give Advice					√		10
Sports					√		8


Full list of particularly important themes which were significantly different across key demographics in Appendix 4.

**Table 3:** Top 10 largest themes which had the highest Chi square values representing greater differentiation between demographic groups by age (younger/older); gender (male/female) and education (no degree / degree and higher)

Theme	Age 64 and under	Age 65 and older	Men	Women	Education: no degree	Education: degree or higher	Chi-square
Working	√						230
Grandchildren		√					69
Cognitive Games		√					62
Running	√						60
Gardening		√					60
Volunteering		√					48
Laughter and Fun	√						46
Golf		√					44
Make People Laugh	√						44
Mountain Sport	√						43
Golf			√				258
Needlework				√			124
Cycling			√				108
Sports			√				108
Sport Watching			√				96
Fishing			√				81
Rational Thinking			√				55
Shopping				√			53
Water Sports			√				42
DIY			√				40
Driving					√		66
Socialising					√		39
Walk Dogs					√		30
Family Connection					√		28
Play Musical Instruments						√	25
Shopping					√		23
Music						√	20
Discuss Literature and Science						√	19
Use Technology						√	19
Traveling						√	19


Full list of largest themes which were significantly different across key demographics in Appendix 5.

Table 2 Top 10 themes selected as particularly important which had the highest Chi square values representing greater differentiation between demographic groups by age (younger/older); gender (male/female) and education (no degree / degree and higher). A full list of particularly important themes which were significantly different across key demographics can be found in Appendix 4.

## Discussion

Building on the scientific foundation provided by previous stages of the ePSOM research programme, we designed and ran a nationwide study with open ended questions to derive free text answers exploring what matters to people about maintaining their brain health within five focus group-derived domains. To our knowledge, this is the first study collecting free and systematically analysing text responses from a very large number of respondents on what is important to them about brain health. The themes and granularity derived from our study are in line with the FDA's guidance for capturing aspects relevant to AD research participants “e.g., [assessing] facility with financial transactions, adequacy of social conversation” (12).

As AD drug development moves to an earlier phase of the neurodegenerative disease spectrum and clinical research targets an earlier, younger population, it is crucial any outcomes are meaningful and relevant to that trial population. Additionally, as upcoming AD treatments are hoped to be disease modifying rather than reducing symptoms, the cognitive domains which respond to the medication may not be the same as with symptomatic treatments measured at a later disease stage (20). We also know from a recent review that lifestyle factors may influence brain health in midlife (21) so it is apposite to examine what matters to people about brain health including lifestyle dependant factors as this will be increasingly relevant in Brain Health Clinics which are developing throughout the UK (16) and Europe (17).

There has been other work collecting evidence on important outcomes focusing on the point of view of people living with dementia (22). The focus of the ePSOM programme though is the maintenance of brain health. As the majority of the individuals in our study had not received a diagnosis of neurodegenerative disease, the findings from our study provide evidence for what matters to people about brain health in normal lived experience which may include people at the earlier (asymptomatic) stages of disease rather than once the dementia syndrome develops. Our findings are supported by literature recognising that AD trials currently do not measure outcomes which are relevant to the patient themselves. Tochel et al. (23) carried out a literature review extracting data from studies where participants described outcomes which matter to them. Their review concluded by demonstrating an array of outcomes which are not commonly captured in clinical trials of new treatments (23).

Changes at the early stages of the AD continuum are currently detected by biomarker assessments, with functional measures used increasingly towards the more symptomatic and advanced stage of the continuum where ultimately impairment is evidenced in basic activities of daily living. However, dementia prevention cohorts have found differences in more than just biomarker assessed pathology, e.g. there is evidence that middle-aged adults at risk of dementia have poorer cognitive performance, principally in visuospatial functions (24) and memory (25). Lau et al. (26) concluded that observing early functional limitations at baseline in the at-risk population had prognostic value in identifying older adults at risk for developing functional disability a few years later (26).

A recent review also concluded that in the pre-dementia stages of AD, executive functions (such as inhibitory abilities), attentional and visuospatial functions can already be impacted (27). A PROM therefore could be viewed as an ecologically valid instrument for cognitive assessment measures which are proxies for what matters to people, especially if the PROM relates to a cognitive process affected early in the course of AD (e.g. activities requiring planning, judgement or navigation/orientation like confidence driving). The key questions here is: if an individual's score changes on a particular domain using a cognitive assessment measure, does this correlate with a change of score in a PROM and is therefore a change meaningful (by definition) to the patient? While functional or Activities of Daily Living scales measure a more direct or practical effect a drug may have, these measures have limitations such as poor psychometric properties (28) and as evidenced by the analysis of key demographic groups in the ePSOM survey, what matters to people about brain health and their function is different depending on age, sex and education levels. By capturing data specific to the individual who in effect derives their own outcome measure, the ePSOM app in development would present an outcome measure for clinical trials that captures changes noticed by and meaningful to the person themselves and therefore more likely to be correlated to their own specific functional outcomes than generic outcomes which were derived by homogenising population level data. Ultimately, employing more meaningful, ecologically valid and sensitive measures will facilitate more drugs to be approved by regulatory bodies which will actually impact on well-being and not just impact on cognition and function ‘on average' between groups (29, 30). Moreover - ePSOMs are immune to cultural, educational and language variability as each outcome is unique to that individual and bears no reference to an external ‘population norm'.

We used an online study design as it was important to allow for free text answers and reach a large number of people. However, this is also a limitation in the study leading to inevitable sampling bias of individuals who are able to access an online survey. There was also a demographic imbalance among the survey respondents with reference to the UK population as a whole, but appropriate analysis focusing on proportions rather than absolute values of this relatively large sample mitigates the effects of the imbalances in the data. The main strength of the study was collecting free text answers and using NLP techniques in the data analysis. Employing NLP techniques to gather evidence for what outcomes matter in AD drug development is unique and we are not aware of any similar studies. Free text answers offer insights which go beyond rating themes on a scale which have been predefined as important by the researchers and are culturally biased and limited. Moreover, the open character of the questions may motivate respondents to reveal more (31). In some regards, our study results may be considered comparable to hundreds of focus group studies, though by using NLP techniques, we are able to extract patterns in answers by key demographic at a scale and level of detail not feasible using traditional qualitative methodologies.

## Conclusion

There is a growing consensus that PROMs should be used in AD trials so that the patient can assess if they observe a change in their well-being which is meaningful and specific to them. Including the patient's perspective is also recommended by regulatory bodies such as the EMA with whom we collaborated in the initial phases of this project, and the FDA. In our study, we included a large number of people collecting free text responses to understand what matters to people about their brain health - our analyses focussed on key demographic groups. This approach is novel in so much as it uses NLP approaches to create a range of outcomes from a theoretically limitless range of possible responses and then can apply these into quantifiable and ecologically valid outcomes. The main criticism and in many ways fatal flaw of current approaches to PROMs is that they are derived at a population level and therefore have to incorporate the characteristics of the population they were derived from. These populations will hold certain language, cultural and ethnic characteristics making their use in other limited in other populations. The ePSOM app will ultimately be used by people in earlier stages of neurodegenerative disease before dementia develops in populations across the globe, in clinical trials with seamless translation into clinical practice.

## Electronic supplementary material

ABOUT THIS SURVEY

The most frequently mentioned themes, broken down by key demographics (full list)

Themes most frequently selected as particularly important, broken down by key demographics (full list)

Statistically significant differences in identifying themes as particularly important, comparing responses from demographic group dyads

Statistically significant differences in identifying themes as particularly important, comparing responses from demographic group dyads

*Funding Sources:* The ePSOM survey was funded by Alzheimer's Research UK.

*Conflicts:* The authors declare no conflict of interest.

*Declarations of interest:* none (all authors).

*Ethical standards:* The study obtained ethics approval from the ACCORD Medical Research Ethics Committee in Edinburgh, Scotland.
