# Supplementary material for: A UK-Wide Study Employing Natural Language Processing to Determine What Matters to People about Brain Health to Improve Drug Development: The Electronic Person-Specific Outcome Measure (ePSOM) Programme
Source: J Prev Alzheimers Dis. 2021 Jun 9;8(4):448–56. doi: 10.14283/jpad.2021.30 (PMC12280764; doi:10.14283/jpad.2021.30)
Supplement: Supplementary file 1 — ABOUT THIS SURVEY [file mmc1.pdf]

[Video introduction]

ABOUT THIS SURVEY

This survey is being carried out by Alzheimer's Research UK and the University of Edinburgh. This page outlines why we are doing this research and what it will involve, to help you decide whether to take part. Please read the following information carefully and discuss it with others if you wish.

What is the project's purpose?

Dementia is a condition caused by brain diseases, most commonly Alzheimer's disease. As these diseases progress, brain cells die and the brain becomes less able to function. As a result, it becomes more difficult for people to carry out everyday tasks. When we talk about dementia, we are talking about declining brain health.

Scientists are working to develop treatments to help people with dementia and even to one day prevent dementia. This survey aims to find out people's views on what they'd like these treatments to help with.

Currently, clinical trials for Alzheimer's and other dementias use two main tools to determine whether a treatment is effective:

- Measuring the effect on cognition (e.g. memory).
- Measuring biological data, such as changes in certain protein levels.

- But these measures do not ne hi cessarily reflect people's perceptions of the effect the drug has on their lives. Diseases like Alzheimer's affect people differently. We want to understand what's most important to you.

Before designing this survey we carried out a focus group study. The findings suggested that the things people valued most fell into five categories:

- Everyday Functioning.
- Enjoying Life.
- Memory Concerns.
- Relationships and Social Connections.
- Sense of Identity.

We've designed this survey to help us explore these themes with many more people. We will ask a series of questions about your brain health. We will also ask you to provide examples of what YOU consider important in your life.

How will you use my answers?

Alzheimer's Research UK will use your answers to inform its policy work in this area. Anonymised data from this survey may also be used to inform the development of a tool to measure whether future clinical trials in Alzheimer's disease offer individuals a real meaningful benefit.

Anonymised data may also be published and shared with other academics and clinicians.

Who would we like to involve?

This is a public survey for anybody aged 18 or over.

What do you want from me?

If you decide to take part, you will be asked for your thoughts about different aspects of your brain health. The survey should take around 30 minutes to complete.

There are no right or wrong answers. Anything you consider important is very valuable for us to hear about. We need a wide range of views to understand what future treatments for dementia should aim to do.

What are the possible benefits of taking part?

Your contribution will help researchers understand what people want from new dementia treatments. Your answers will also help inform healthcare decision-makers who assess future treatments. Your contribution is very valuable.

What if something goes wrong?

If you want to complain about any aspect of the study, contact CDP@ed.ac.uk and we will respond immediately.

Some people may feel uncomfortable thinking about different aspects of their brain health. Remember – you are free to stop completing the survey at any time.

Will anyone know I'm taking part in this project?

All your responses to this survey will be anonymous. You will not be identified.

What will happen to the results?

We will produce a report on the results, which we will publish on Alzheimer's Research UK's website. We will also publish the results in academic journals and present them at conferences. You will not be identified in any reports or publications.

Who is organising and funding the research?

Alzheimer's Research UK, the UK's leading dementia research charity.

The team organising this activity are:

- At the University of Edinburgh: Prof Craig Ritchie, Dr Graciela Muniz-Terrera, Dr Saturnino Luz and Stina Saunders.
- At Alzheimer's Research UK: Dr Alison Evans.

Who has ethically reviewed the project?

ACCORD Medical Research Ethics Committee has reviewed and approved the project.

Privacy notice

Please do not provide any personal and/or sensitive data in this survey, such as your name or political persuasion. If you include personal and/or sensitive data in your answers, the data holders will delete it before analysis.

By taking part in the survey you are agreeing with the following statements:

1) I have read and understand the information about this project.

2) I am aged 18 or over.

3) I understand that my participation in this survey is voluntary and that I am free to withdraw at any time.

4) I give permission for the project's researchers to access my anonymised responses.

☐ I agree to take part in this survey

[Start survey]

About your brain health

Dementia is a neurodegenerative condition caused by underlying diseases in the brain. The most common cause of dementia is Alzheimer's disease.

Over time, brain cells die and the brain becomes less able to function properly. This is why when we talk about dementia we are also talking about brain health.

Please answer these questions about your brain health and what you do to maintain it.

On a scale of 1-10 how would you rate your current brain health?

1 means you think you are not at all capable of carrying out day to day activities and interactions with others.

10 means you think you are fully capable of undertaking day to day activities and interactions with others.

1 ☐ 2 ☐ 3 ☐ 4 ☐ 5 ☐ 6 ☐ 7 ☐ 8 ☐ 9 ☐ 10 ☐

Have you ever told anyone, including a friend or family member, that you are worried about your brain health?

☐ Yes

☐ No

☐ Don't know

Do you do anything specifically to maintain brain health? These are activities that you purposefully do in order to maintain your brain health.

☐ I keep physically active

☐ I eat a healthy diet

☐ I watch my weight

☐ I try to keep my mind active

☐ I try to keep socially active

☐ I try not to smoke

☐ I try not to drink excess alcohol

☐ I look after my mental health (stress, mood)

☐ Other:___________

Understanding what treatment outcomes would be most important to you

When people develop diseases that affect brain health, their ability to undertake day to day activities can change.

In this next section, we will ask a series of questions about what you feel would be most important to still be able to do, if your brain health deteriorated.

We'll be asking about five categories:

- Everyday functioning
- Enjoying life
- Memory concerns
- Relationships and Social interactions
- Sense of identity

We'd like you to answer as honestly and fully as possible. Don't worry about repeating things that are important to you in multiple categories if they overlap.

Everyday functioning

Please list everyday activities that you are able to do now that would be important for you to be able to continue doing if your brain health deteriorated. These can be things that pop into your head – they can be simple or complex.

An example could be '*I would like to be able to still drive my car*'.

Please provide at least one example.

I would like to be able to:

[Enter your answer here]

Enjoyable activities

Please list hobbies or social/lifestyle activities that you do now and would be important for you to retain if your brain health deteriorated. It can be anything at all you enjoy doing.

An example of this could be '*I would like to be able to play golf''*.

Please provide at least one example.

I would like to be able to:

[Enter your answer here]

Relationships and social interactions

Please think about how you are with other people, like friends or family. What abilities would you want to protect that are important for helping you maintain meaningful relationships? It can be anything that relates to other people around you.

An example could be '*I would like to be able to chat with friends'*.

Please provide at least one example.

I would like to be able to:

[Enter your answer here]

Thinking abilities

Thinking abilities are a way of describing how we are able to think. For instance, this could include making decisions, planning ahead, remembering, etc. Considering your current thinking abilities, which abilities would be the most important to still be able to do if your brain health deteriorated?

An example could be *'I would like to be able to understand the storyline in a film'*.

Please provide at least one example.

I would like to be able to:

[Enter your answer here]

Sense of who you are as a person

Now think about who you are and what is central to making you feel like you and giving you a sense of purpose. Please consider which aspects of your sense of identity would be important for you to still have if your brain health got worse.

An example could be *'I want to still be able to give advice to my family and friends'*.

Please provide at least one example.

I would like to be able to:

[Enter your answer here]

Other important activities or abilities

There may be some other important aspects of your brain health that we have not asked about. If you would like to detail anything else that is important to you, please do so in the box below

[Enter your answer here]

What would be the most important to you?

You have now listed the abilities and activities that are the most important to you to maintain if your brain health deteriorated. Which activities or abilities listed below are most important to you overall?

Select up to five, starting with the most important one.

1 [- Select one -]

2 [- Select one -]

3 [- Select one -]

4 [- Select one -]

5 [- Select one -]

About your health

Thank you for answering our question on what matters to you about your brain health. In this section, we're asking about your health and medical history.

Have ever seen a doctor about your brain health?

☐ Yes

☐ No

☐ Don’t know

If yes, which doctor did you see?

☐ GP

☐ Psychiatrist in a Memory Clinic

☐ Psychiatrist outside a Memory Clinic

☐ Neurologist

☐ Geriatrician

☐ Other

☐ Not sure

Have you ever been given a diagnosis related to your brain health?

☐ Yes

☐ No

☐ Prefer not to say

If yes, what was the diagnosis you were given?

☐ Mild Cognitive Impairment

☐ Alzheimer’s disease

☐ Mixed dementia

☐ Dementia with Lewy bodies

☐ Frontotemporal dementia

☐ Vascular dementia

☐ Parkinson’s disease dementia

☐ Parkinson’s disease

☐ Huntington’s disease

☐ Creutzfeldt-Jakob Disease

☐ Korsakoff Syndrome

☐ Motor neurone disease

☐ Subjective Cognitive Disorder

☐ Other (please list) ____________________

☐ Not sure

☐ Prefer not to say

If yes, do you currently take or have you ever taken any prescribed medication for this condition?

☐ Yes

☐ No

☐ Prefer not to say

If yes, which of these do you take:

☐ Aricept, also known as Donepezil

☐ Exelon, also known as Rivastigmine

☐ Galanthamine, also known as Reminyl

☐ Memantine, also known as Namenda

☐Other [   ]

Do you take any non-prescribed medication for brain health?

☐ No

☐Yes

If yes, which of these do you take:

☐ Vitamin Supplements (please list)¬___________________

☐ Gingko Biloba

☐ Souvenaid

☐ Other (please list) ______________________

☐ Prefer not to say

***

About you

What is your age?

[Enter your answer here]

What is your gender?

☐ Male

☐Female

☐Other ________________________

☐Prefer not to say

What is your current work situation?

☐Full time paid work

☐Part time paid work

☐ Self-employed

☐Volunteer

☐Not working

☐Retired

What is your highest level of education?

☐Postgraduate degree

☐Degree or equivalent

☐ A Level or equivalent

☐Secondary school of equivalent

☐No qualifications

☐Other

☐Prefer not to say

What is your current living situation?

☐Home owner

☐Renting

☐Live with family or friends

☐Live alone

☐Other

☐Prefer not to say

Where do you live?

☐Urban area

☐Rural area

☐Prefer not to say

What is your marital status?

☐Married

☐Single

☐Divorced

☐Widowed

☐ Prefer not to say

Smoking Status

☐ Current smoker

☐ Heavy

☐ Moderate

☐ Occasional

☐ Ex-smoker

☐ Never smoked

Which option below best describes your alcohol intake?

☐I drink every day of the week

☐I drink most days of the week

☐I drink a few times a month

☐I drink only a few times a year

☐I don't drink at all

Have you ever supported a relative, friend or neighbour with dementia?

☐ Yes

☐ No

☐ Don't know

Did someone assist you in completing this survey today?

☐ Yes

☐ No
