# Supplementary material for: A UK-Wide Study Employing Natural Language Processing to Determine What Matters to People about Brain Health to Improve Drug Development: The Electronic Person-Specific Outcome Measure (ePSOM) Programme
Source: J Prev Alzheimers Dis. 2021 Jun 9;8(4):448–56. doi: 10.14283/jpad.2021.30 (PMC12280764; doi:10.14283/jpad.2021.30)
Supplement: Supplementary file 2 — The most frequently mentioned themes, broken down by key demographics (full list) [file mmc2.pdf]

The most frequently mentioned themes, broken down by key demographics (full list)

	Theme	Male	%	Female	%	Age <= 64	%	Age >= 65	%	No Degree	%	Degree	%
1	Reading	598	3.62	2806	4.27	2158	4.12	1259	4.18	1028	3.93	2389	4.24
2	Driving	678	4.10	2212	3.37	1734	3.31	1161	3.85	1117	4.28	1778	3.15
3	Friendships	438	2.65	2399	3.65	1729	3.30	1116	3.70	918	3.51	1927	3.42
4	Follow A Storyline	465	2.81	2215	3.37	1815	3.47	873	2.90	803	3.07	1885	3.34
5	Socialising	533	3.22	2054	3.12	1669	3.19	922	3.06	967	3.70	1624	2.88
6	Family Connection	500	3.03	2049	3.12	1664	3.18	892	2.96	934	3.57	1622	2.88
7	Walking	558	3.38	1875	2.85	1523	2.91	917	3.04	768	2.94	1672	2.97
8	Conversation And Chat	338	2.05	1500	2.28	1219	2.33	627	2.08	613	2.35	1233	2.19
9	Use Technology	351	2.12	1376	2.09	1043	1.99	689	2.29	464	1.78	1268	2.25
10	Mix Family Connections Friendships	322	1.95	1387	2.11	1143	2.18	568	1.88	597	2.28	1114	1.98
11	Cooking	232	1.40	1439	2.19	1188	2.27	490	1.63	472	1.81	1206	2.14
12	Gardening	285	1.72	1324	2.01	877	1.67	740	2.46	523	2.00	1094	1.94
13	Cognitive Games	261	1.58	956	1.45	641	1.22	577	1.91	431	1.65	787	1.40
14	Maintain Independence	180	1.09	917	1.40	727	1.39	373	1.24	372	1.42	728	1.29
15	Manage Finances	231	1.40	825	1.26	587	1.12	472	1.57	318	1.22	741	1.31
16	Meaningful Conversations	219	1.33	811	1.23	692	1.32	341	1.13	350	1.34	683	1.21
17	Shopping	113	0.68	915	1.39	695	1.33	333	1.10	398	1.52	630	1.12
18	Dining	133	0.80	860	1.31	688	1.31	307	1.02	287	1.10	708	1.26
19	Make Decisions	135	0.82	826	1.26	675	1.29	289	0.96	288	1.10	676	1.20
20	Feel Wanted And Needed	202	1.22	715	1.09	600	1.15	321	1.06	292	1.12	629	1.12
21	Recognise People	176	1.06	718	1.09	617	1.18	280	0.93	313	1.20	584	1.04
22	Personal Attributes And Social Skills	176	1.06	649	0.99	513	0.98	314	1.04	284	1.09	543	0.96
23	Travelling	180	1.09	629	0.96	494	0.94	318	1.06	228	0.87	584	1.04
24	Music	195	1.18	611	0.93	512	0.98	298	0.99	197	0.75	613	1.09
25	Volunteering	174	1.05	599	0.91	398	0.76	376	1.25	227	0.87	547	0.97
26	Personal Hygiene	99	0.60	672	1.02	563	1.08	208	0.69	239	0.91	532	0.94
27	Mixtheatrecinema	91	0.55	653	0.99	487	0.93	260	0.86	204	0.78	543	0.96
28	Remember Past	162	0.98	580	0.88	505	0.96	238	0.79	234	0.90	509	0.90
29	Grandchildren	137	0.83	578	0.88	347	0.66	369	1.22	260	1.00	456	0.81
30	Communicate Effectively	178	1.08	489	0.74	359	0.69	310	1.03	228	0.87	441	0.78
31	Analyse And Solve Problems	153	0.93	507	0.77	418	0.80	249	0.83	191	0.73	476	0.84
32	Give Advice	185	1.12	473	0.72	444	0.85	215	0.71	238	0.91	421	0.75
33	Help Others	141	0.85	513	0.78	409	0.78	247	0.82	253	0.97	403	0.71
34	Exercise	143	0.87	492	0.75	431	0.82	204	0.68	197	0.75	438	0.78

35	Walk Dogs	80	0.48	537	0.82	470	0.90	150	0.50	261	1.00	359	0.64
36	Swimming	102	0.62	511	0.78	465	0.89	153	0.51	189	0.72	429	0.76
37	Planning And Organising Skills	98	0.59	517	0.79	387	0.74	228	0.76	161	0.62	454	0.81
38	Support Family	84	0.51	524	0.80	419	0.80	193	0.64	197	0.75	415	0.74
39	Needlework	12	0.07	597	0.91	384	0.73	226	0.75	202	0.77	408	0.72
40	Understand Current Affairs	150	0.91	440	0.67	358	0.68	232	0.77	156	0.60	434	0.77
41	Cycling	218	1.32	364	0.55	429	0.82	154	0.51	164	0.63	419	0.74
42	Working	87	0.53	475	0.72	534	1.02	33	0.11	190	0.73	377	0.67
43	Going On Holidays	96	0.58	455	0.69	348	0.66	204	0.68	205	0.78	347	0.62
44	Watch Tv	117	0.71	416	0.63	366	0.70	173	0.57	159	0.61	380	0.67
45	Live At Home	81	0.49	441	0.67	330	0.63	193	0.64	144	0.55	379	0.67
46	Sense Of Humour	94	0.57	394	0.60	365	0.70	126	0.42	155	0.59	336	0.60
47	Good Listener	70	0.42	407	0.62	310	0.59	170	0.56	134	0.51	346	0.61
48	Household Chores	60	0.36	415	0.63	307	0.59	169	0.56	156	0.60	320	0.57
49	Remember Peoples Name	128	0.77	341	0.52	273	0.52	196	0.65	155	0.59	314	0.56
50	Leisure Travel	96	0.58	363	0.55	269	0.51	191	0.63	135	0.52	325	0.58
51	Plan Future	87	0.53	362	0.55	317	0.61	134	0.44	128	0.49	323	0.57
52	Rational Thinking	154	0.93	296	0.45	265	0.51	185	0.61	114	0.44	336	0.60
53	Hobbies	102	0.62	339	0.52	259	0.49	185	0.61	157	0.60	287	0.51
54	Remember Important Dates	49	0.30	362	0.55	294	0.56	118	0.39	130	0.50	282	0.50
55	Capacity To Understand Text	79	0.48	325	0.49	272	0.52	132	0.44	129	0.49	275	0.49
56	Religious Participation	66	0.40	333	0.51	217	0.41	183	0.61	104	0.40	296	0.52
57	Writing	74	0.45	323	0.49	259	0.49	139	0.46	105	0.40	293	0.52
58	Singing	40	0.24	347	0.53	202	0.39	187	0.62	98	0.38	291	0.52
59	Pets	56	0.34	332	0.51	286	0.55	103	0.34	98	0.38	291	0.52
60	Take Part In Activities	80	0.48	300	0.46	201	0.38	182	0.60	97	0.37	286	0.51
61	Analytical Skills	101	0.61	270	0.41	227	0.43	145	0.48	92	0.35	280	0.50
62	Confidence	80	0.48	274	0.42	205	0.39	150	0.50	93	0.36	262	0.46
63	Gym	76	0.46	273	0.42	253	0.48	96	0.32	128	0.49	221	0.39
64	Caring Responsibilities	67	0.41	279	0.42	179	0.34	168	0.56	130	0.50	217	0.38
65	Ability To Learn New Skills	84	0.51	256	0.39	225	0.43	118	0.39	87	0.33	256	0.45
66	Running	86	0.52	239	0.36	275	0.53	51	0.17	88	0.34	238	0.42
67	Feel Valued And Self-Worth	81	0.49	243	0.37	196	0.37	129	0.43	93	0.36	232	0.41
68	Sports	141	0.85	184	0.28	193	0.37	132	0.44	133	0.51	192	0.34
69	Mental Agility	103	0.62	219	0.33	186	0.36	137	0.45	94	0.36	229	0.41
70	Dressing Style	23	0.14	297	0.45	246	0.47	74	0.25	101	0.39	219	0.39
71	Use Toilet	46	0.28	248	0.38	207	0.40	89	0.30	80	0.31	216	0.38
72	Play Musical Instruments	83	0.50	205	0.31	191	0.36	97	0.32	51	0.20	237	0.42
73	Spouse	62	0.38	217	0.33	195	0.37	85	0.28	97	0.37	183	0.32
74	Maintain Dignity	64	0.39	194	0.30	165	0.32	94	0.31	78	0.30	181	0.32
75	Mountain Sports	60	0.36	186	0.28	207	0.40	40	0.13	58	0.22	189	0.34
76	Staying Active	77	0.47	168	0.26	141	0.27	105	0.35	80	0.31	166	0.29
77	Make People Laugh	48	0.29	195	0.30	206	0.39	39	0.13	64	0.24	181	0.32
78	Golf	150	0.91	94	0.14	104	0.20	140	0.46	96	0.37	148	0.26
79	Plan Leisure Time	47	0.28	193	0.29	170	0.32	70	0.23	69	0.26	171	0.30
80	Artwork	38	0.23	200	0.30	138	0.26	101	0.34	72	0.28	167	0.30

81	Yoga	11	0.07	227	0.35	164	0.31	74	0.25	73	0.28	165	0.29
82	Role In The Community	59	0.36	174	0.26	118	0.23	115	0.38	57	0.22	176	0.31
83	Follow A Conversation	32	0.19	193	0.29	158	0.30	69	0.23	65	0.25	162	0.29
84	Express Opinions	45	0.27	176	0.27	135	0.26	88	0.29	66	0.25	157	0.28
85	Dancing	15	0.09	206	0.31	155	0.30	67	0.22	68	0.26	154	0.27
86	Plan Holidays	30	0.18	189	0.29	140	0.27	81	0.27	67	0.26	154	0.27
87	Laughter And Fun	29	0.18	183	0.28	185	0.35	30	0.10	71	0.27	144	0.26
88	Debate Politics	67	0.41	147	0.22	109	0.21	105	0.35	42	0.16	172	0.31
89	Coffee With Friends	16	0.10	196	0.30	137	0.26	75	0.25	62	0.24	150	0.27
90	Empathy	38	0.23	171	0.26	139	0.27	72	0.24	59	0.23	152	0.27
91	Mixcookingbaking	23	0.14	164	0.25	133	0.25	54	0.18	58	0.22	129	0.23
92	Kind And Caring	41	0.25	145	0.22	132	0.25	54	0.18	51	0.20	135	0.24
93	Contribute To Family	44	0.27	140	0.21	97	0.19	87	0.29	59	0.23	125	0.22
94	Craftwork	27	0.16	157	0.24	108	0.21	76	0.25	61	0.23	123	0.22
95	Traveling	37	0.22	142	0.22	121	0.23	58	0.19	29	0.11	150	0.27
96	Mixboard Games And Cards	55	0.33	122	0.19	70	0.13	107	0.35	61	0.23	116	0.21
97	Look Good	25	0.15	151	0.23	98	0.19	78	0.26	74	0.28	102	0.18
98	Follow Tv And News	49	0.30	126	0.19	106	0.20	70	0.23	44	0.17	132	0.23
99	Remember Names	39	0.24	134	0.20	90	0.17	83	0.28	58	0.22	115	0.20
100	Remember Where Put Things	38	0.23	134	0.20	108	0.21	64	0.21	64	0.24	108	0.19
101	Plan Family Life	33	0.20	138	0.21	104	0.20	68	0.23	74	0.28	98	0.17
102	Intelligence	54	0.33	115	0.17	91	0.17	78	0.26	47	0.18	122	0.22
103	Creative Activities	35	0.21	124	0.19	84	0.16	77	0.26	38	0.15	123	0.22
104	Mixgalleriesmuseums	29	0.18	125	0.19	94	0.18	61	0.20	35	0.13	120	0.21
105	Remember Books	31	0.19	124	0.19	110	0.21	45	0.15	60	0.23	95	0.17
106	Mixconcertsfestivals	18	0.11	135	0.21	89	0.17	64	0.21	30	0.11	123	0.22
107	Take Care Of Things	12	0.07	137	0.21	106	0.20	43	0.14	48	0.18	101	0.18
108	Remember Day To Day Things	22	0.13	125	0.19	98	0.19	49	0.16	55	0.21	92	0.16
109	Concentrate And Understand Books	45	0.27	101	0.15	78	0.15	68	0.23	35	0.13	111	0.20
110	Use Public Transport	20	0.12	123	0.19	99	0.19	47	0.16	39	0.15	107	0.19
111	Contribute To Conversation	41	0.25	102	0.16	85	0.16	59	0.20	29	0.11	115	0.20
112	Academic Activities	42	0.25	101	0.15	100	0.19	43	0.14	24	0.09	119	0.21
113	Understand Tv Programmes	25	0.15	116	0.18	77	0.15	64	0.21	52	0.20	89	0.16
114	Prioritise And Analyse Tasks	39	0.24	96	0.15	85	0.16	50	0.17	39	0.15	96	0.17
115	Assess Complex Issues	48	0.29	83	0.13	86	0.16	47	0.16	36	0.14	97	0.17
116	Exercise Classes	8	0.05	120	0.18	81	0.15	47	0.16	42	0.16	86	0.15
117	Gaming	35	0.21	89	0.14	89	0.17	36	0.12	38	0.15	87	0.15
118	Remember Recent Events	29	0.18	96	0.15	86	0.16	39	0.13	35	0.13	90	0.16
119	Getting Dressed	19	0.11	105	0.16	96	0.18	28	0.09	42	0.16	82	0.15
120	Share Memories	20	0.12	102	0.16	84	0.16	38	0.13	27	0.10	95	0.17
121	Pub	43	0.26	69	0.10	83	0.16	29	0.10	35	0.13	77	0.14
122	Mixgardeningother	27	0.16	84	0.13	60	0.11	51	0.17	39	0.15	72	0.13
123	Racket Sports	30	0.18	80	0.12	72	0.14	38	0.13	29	0.11	81	0.14
124	Baking	1	0.01	108	0.16	94	0.18	15	0.05	33	0.13	76	0.13
125	Same Person	22	0.13	85	0.13	66	0.13	42	0.14	39	0.15	69	0.12
126	Group Activities	24	0.15	83	0.13	47	0.09	60	0.20	29	0.11	78	0.14

127	Discuss Literature And Science	30	0.18	75	0.11	64	0.12	42	0.14	12	0.05	94	0.17
128	Situational Awareness	24	0.15	82	0.12	75	0.14	31	0.10	28	0.11	78	0.14
129	Organise Home	16	0.10	89	0.14	65	0.12	40	0.13	41	0.16	64	0.11
130	Academic Activites	29	0.18	74	0.11	59	0.11	44	0.15	18	0.07	85	0.15
131	Control Budgets	14	0.08	89	0.14	67	0.13	36	0.12	45	0.17	58	0.10
132	Mixconcertstheatrecinemamuseums	12	0.07	89	0.14	43	0.08	58	0.19	19	0.07	82	0.15
133	Plan Meals	7	0.04	89	0.14	64	0.12	33	0.11	38	0.15	59	0.10
134	Remembering Words	25	0.15	72	0.11	72	0.14	25	0.08	32	0.12	65	0.12
135	Make Tea	8	0.05	83	0.13	75	0.14	19	0.06	33	0.13	61	0.11
136	Happy And Loved	17	0.10	76	0.12	68	0.13	26	0.09	33	0.13	61	0.11
137	Follow Instructions	15	0.09	76	0.12	52	0.10	40	0.13	32	0.12	60	0.11
138	Water Sports	44	0.27	48	0.07	53	0.10	39	0.13	32	0.12	60	0.11
139	Follow A Recipe	4	0.02	85	0.13	68	0.13	21	0.07	32	0.12	57	0.10
140	Sport Watching	55	0.33	33	0.05	71	0.14	17	0.06	30	0.11	58	0.10
141	Follow Needlework Pattern	4	0.02	81	0.12	62	0.12	23	0.08	29	0.11	56	0.10
142	Remember Routes	26	0.16	52	0.08	47	0.09	31	0.10	25	0.10	53	0.09
143	Wit And Humour	20	0.12	54	0.08	49	0.09	27	0.09	14	0.05	62	0.11
144	Read Music	17	0.10	57	0.09	44	0.08	30	0.10	20	0.08	54	0.10
145	Manage Appointments	6	0.04	64	0.10	42	0.08	29	0.10	22	0.08	49	0.09
146	New Friends	14	0.08	56	0.09	44	0.08	26	0.09	18	0.07	52	0.09
147	Run Household	16	0.10	53	0.08	43	0.08	26	0.09	28	0.11	41	0.07
148	Maintain Contact	11	0.07	56	0.09	38	0.07	29	0.10	25	0.10	42	0.07
149	Speak Foreign Languages	15	0.09	50	0.08	36	0.07	29	0.10	10	0.04	55	0.10
150	Mixcinematheatre	12	0.07	51	0.08	38	0.07	26	0.09	21	0.08	43	0.08
151	Remember Love	16	0.10	46	0.07	43	0.08	19	0.06	34	0.13	28	0.05
152	Mix Love Other	5	0.03	55	0.08	44	0.08	17	0.06	18	0.07	43	0.08
153	Remember Conversations	10	0.06	50	0.08	36	0.07	24	0.08	21	0.08	39	0.07
154	Listen To Radio	12	0.07	47	0.07	29	0.06	30	0.10	17	0.07	42	0.07
155	Pilates	3	0.02	53	0.08	29	0.06	27	0.09	13	0.05	43	0.08
156	Entertain	8	0.05	47	0.07	25	0.05	30	0.10	19	0.07	36	0.06
157	Dancing	4	0.02	47	0.07	29	0.06	24	0.08	20	0.08	33	0.06
158	Woman'S Role In Family	8	0.05	45	0.07	35	0.07	18	0.06	19	0.07	34	0.06
159	Not Be A Burden	14	0.08	39	0.06	30	0.06	23	0.08	23	0.09	30	0.05
160	Manage Diary	7	0.04	43	0.07	31	0.06	19	0.06	10	0.04	40	0.07
161	Retain A Good Memory	20	0.12	29	0.04	24	0.05	25	0.08	20	0.08	29	0.05
162	Remember Peoples Name And Face	14	0.08	34	0.05	29	0.06	19	0.06	11	0.04	37	0.07
163	Remain Positive	6	0.04	35	0.05	30	0.06	12	0.04	6	0.02	36	0.06
164	Mixridinghorseridingbike	9	0.05	31	0.05	34	0.06	7	0.02	16	0.06	25	0.04
165	Contribute To Society	12	0.07	28	0.04	25	0.05	15	0.05	4	0.02	36	0.06
166	Mixyogapilates	3	0.02	36	0.05	21	0.04	18	0.06	14	0.05	25	0.04
167	Photography	15	0.09	23	0.03	27	0.05	11	0.04	10	0.04	28	0.05
168	Sex Life	17	0.10	18	0.03	23	0.04	13	0.04	9	0.03	27	0.05
169	In Control Of Life	12	0.07	24	0.04	24	0.05	12	0.04	11	0.04	25	0.04
170	Motherhood	1	0.01	35	0.05	29	0.06	7	0.02	12	0.05	24	0.04
171	Winter Sports	14	0.08	22	0.03	27	0.05	9	0.03	10	0.04	26	0.05
172	Bowls	15	0.09	20	0.03	7	0.01	28	0.09	19	0.07	16	0.03

173	Tai Chi	3	0.02	30	0.05	15	0.03	18	0.06	9	0.03	24	0.04
174	Diy	21	0.13	10	0.02	16	0.03	16	0.05	8	0.03	24	0.04
175	Good Company	7	0.04	19	0.03	18	0.03	8	0.03	8	0.03	18	0.03
176	Fishing	23	0.14	1	0.00	16	0.03	8	0.03	13	0.05	11	0.02
177	Play Games	6	0.04	16	0.02	18	0.03	4	0.01	8	0.03	14	0.02
178	Staying Safe	1	0.01	18	0.03	18	0.03	2	0.01	6	0.02	14	0.02
179	Mental Arithmetic	10	0.06	10	0.02	11	0.02	9	0.03	3	0.01	17	0.03
180	Retain Maths Ability	4	0.02	14	0.02	13	0.02	5	0.02	1	0.00	17	0.03
181	Camping	7	0.04	8	0.01	11	0.02	4	0.01	2	0.01	13	0.02
182	Hearing	4	0.02	3	0.00	4	0.01	3	0.01	3	0.01	4	0.01
183	Compassionate	2	0.01	5	0.01	7	0.01	0	0.00	2	0.01	5	0.01
184	Not Elsewhere Categorised	1463	8.85	4539	6.91	3449	6.59	2566	8.51	1934	7.40	4081	7.24
	Total	16528	100.00	65731	100.00	52372	100.00	30142	100.00	26128	100.00	56386	100.00
