# Supplementary material for: A UK-Wide Study Employing Natural Language Processing to Determine What Matters to People about Brain Health to Improve Drug Development: The Electronic Person-Specific Outcome Measure (ePSOM) Programme
Source: J Prev Alzheimers Dis. 2021 Jun 9;8(4):448–56. doi: 10.14283/jpad.2021.30 (PMC12280764; doi:10.14283/jpad.2021.30)
Supplement: Supplementary file 3 — Themes most frequently selected as particularly important, broken down by key demographics (full list) [file mmc3.pdf]

Themes most frequently selected as particularly important, broken down by key demographics (full list)

	Theme	Male	%	Female	%	Age <= 64	%	Age >= 65	%	No Degree	%	Degree	%
1	Family Connection	280	4.51	988	4.74	866	5.06	407	4.08	516	5.25	757	4.38
2	Driving	330	5.31	936	4.49	707	4.13	559	5.60	568	5.78	698	4.04
3	Socialising	246	3.96	845	4.06	694	4.05	398	3.99	452	4.60	640	3.70
4	Reading	226	3.64	850	4.08	658	3.84	419	4.20	344	3.50	733	4.24
5	Friendships	189	3.04	804	3.86	606	3.54	388	3.89	349	3.55	645	3.73
6	Walking	211	3.40	600	2.88	505	2.95	309	3.09	292	2.97	522	3.02
7	Maintain Independence	119	1.92	642	3.08	510	2.98	252	2.52	277	2.82	485	2.81
8	Mix Family Connections Friendships	145	2.33	545	2.62	473	2.76	217	2.17	264	2.69	426	2.47
9	Conversation And Chat	129	2.08	505	2.42	430	2.51	207	2.07	232	2.36	405	2.34
10	Follow A Storyline	123	1.98	401	1.93	348	2.03	177	1.77	169	1.72	356	2.06
11	Personal Hygiene	66	1.06	437	2.10	372	2.17	131	1.31	165	1.68	338	1.96
12	Cooking	87	1.40	390	1.87	342	2.00	136	1.36	143	1.46	335	1.94
13	Use Technology	111	1.79	343	1.65	247	1.44	207	2.07	152	1.55	302	1.75
14	Recognise People	100	1.61	334	1.60	313	1.83	121	1.21	160	1.63	274	1.59
15	Make Decisions	66	1.06	349	1.68	298	1.74	117	1.17	133	1.35	282	1.63
16	Meaningful Conversations	104	1.67	298	1.43	278	1.62	124	1.24	150	1.53	252	1.46
17	Feel Wanted And Needed	99	1.59	296	1.42	255	1.49	141	1.41	127	1.29	269	1.56
18	Gardening	65	1.05	306	1.47	184	1.07	190	1.90	137	1.39	237	1.37
19	Manage Finances	88	1.42	275	1.32	194	1.13	169	1.69	134	1.36	229	1.33
20	Communicate Effectively	89	1.43	240	1.15	176	1.03	153	1.53	122	1.24	207	1.20
21	Live At Home	53	0.85	255	1.22	186	1.09	123	1.23	98	1.00	211	1.22
22	Grandchildren	63	1.01	222	1.07	150	0.88	135	1.35	115	1.17	170	0.98
23	Exercise	67	1.08	214	1.03	195	1.14	86	0.86	94	0.96	187	1.08
24	Walk Dogs	42	0.68	229	1.10	204	1.19	68	0.68	129	1.31	143	0.83
25	Volunteering	62	1.00	199	0.96	136	0.79	125	1.25	73	0.74	188	1.09
26	Personal Attributes And Social Skills	58	0.93	201	0.97	166	0.97	93	0.93	95	0.97	164	0.95
27	Remember Peoples Name	73	1.17	166	0.80	139	0.81	100	1.00	95	0.97	144	0.83
28	Remember Past	59	0.95	178	0.85	163	0.95	75	0.75	83	0.84	155	0.90
29	Support Family	38	0.61	199	0.96	164	0.96	74	0.74	84	0.85	154	0.89
30	Music	80	1.29	150	0.72	141	0.82	90	0.90	62	0.63	169	0.98
31	Travelling	55	0.89	173	0.83	138	0.81	92	0.92	71	0.72	159	0.92
32	Use Toilet	33	0.53	176	0.84	149	0.87	61	0.61	63	0.64	147	0.85

33	Sense Of Humour	40	0.64	152	0.73	144	0.84	50	0.50	76	0.77	118	0.68
34	Pets	29	0.47	162	0.78	152	0.89	39	0.39	50	0.51	141	0.82
35	Shopping	21	0.34	168	0.81	127	0.74	62	0.62	91	0.93	98	0.57
36	Working	38	0.61	150	0.72	176	1.03	13	0.13	74	0.75	115	0.67
37	Help Others	48	0.77	140	0.67	116	0.68	72	0.72	85	0.87	103	0.60
38	Cognitive Games	56	0.90	131	0.63	77	0.45	110	1.10	77	0.78	110	0.64
39	Hobbies	39	0.63	143	0.69	103	0.60	79	0.79	67	0.68	115	0.67
40	Dining	32	0.52	143	0.69	122	0.71	54	0.54	63	0.64	113	0.65
41	Give Advice	59	0.95	110	0.53	107	0.63	63	0.63	82	0.83	88	0.51
42	Caring Responsibilities	38	0.61	132	0.63	84	0.49	86	0.86	76	0.77	94	0.54
43	Household Chores	24	0.39	139	0.67	105	0.61	59	0.59	50	0.51	114	0.66
44	Analyse And Solve Problems	37	0.60	122	0.59	102	0.60	58	0.58	59	0.60	101	0.58
45	Maintain Dignity	39	0.63	116	0.56	95	0.55	60	0.60	56	0.57	99	0.57
46	Going On Holidays	32	0.52	120	0.58	97	0.57	55	0.55	75	0.76	77	0.45
47	Spouse	36	0.58	115	0.55	108	0.63	43	0.43	45	0.46	106	0.61
48	Staying Active	52	0.84	90	0.43	78	0.46	64	0.64	46	0.47	96	0.56
49	Religious Participation	21	0.34	119	0.57	79	0.46	61	0.61	42	0.43	98	0.57
50	Look Good	22	0.35	116	0.56	78	0.46	60	0.60	62	0.63	76	0.44
51	Cycling	58	0.93	79	0.38	102	0.60	35	0.35	50	0.51	87	0.50
52	Understand Current Affairs	47	0.76	84	0.40	86	0.50	45	0.45	41	0.42	90	0.52
53	Rational Thinking	47	0.76	83	0.40	86	0.50	44	0.44	46	0.47	84	0.49
54	Needlework	2	0.03	120	0.58	80	0.47	43	0.43	60	0.61	63	0.36
55	Planning And Organising Skills	20	0.32	93	0.45	70	0.41	43	0.43	37	0.38	76	0.44
56	Good Listener	20	0.32	91	0.44	76	0.44	36	0.36	34	0.35	78	0.45
57	Singing	16	0.26	96	0.46	56	0.33	56	0.56	35	0.36	77	0.45
58	Feel Valued And Self-Worth	24	0.39	83	0.40	72	0.42	36	0.36	34	0.35	74	0.43
59	Leisure Travel	26	0.42	81	0.39	55	0.32	52	0.52	38	0.39	69	0.40
60	Plan Future	22	0.35	77	0.37	73	0.43	26	0.26	29	0.30	70	0.41
61	Confidence	20	0.32	74	0.36	55	0.32	39	0.39	26	0.26	68	0.39
62	Watch Tv	31	0.50	61	0.29	52	0.30	41	0.41	30	0.31	63	0.36
63	Sports	49	0.79	44	0.21	49	0.29	44	0.44	48	0.49	45	0.26
64	Mental Agility	32	0.52	60	0.29	59	0.34	34	0.34	36	0.37	57	0.33
65	Running	32	0.52	60	0.29	77	0.45	15	0.15	30	0.31	62	0.36
66	Take Part In Activities	20	0.32	71	0.34	53	0.31	39	0.39	30	0.31	62	0.36
67	Traveling	23	0.37	68	0.33	61	0.36	30	0.30	17	0.17	74	0.43
68	Capacity To Understand Text	21	0.34	68	0.33	54	0.32	35	0.35	25	0.25	64	0.37
69	Swimming	16	0.26	70	0.34	64	0.37	25	0.25	38	0.39	51	0.30
70	Ability To Learn New Skills	24	0.39	62	0.30	54	0.32	32	0.32	25	0.25	61	0.35
71	Mixtheatrecinema	12	0.19	71	0.34	52	0.30	32	0.32	27	0.27	57	0.33
72	Writing	24	0.39	59	0.28	48	0.28	35	0.35	21	0.21	62	0.36
73	Analytical Skills	28	0.45	53	0.25	46	0.27	35	0.35	24	0.24	57	0.33
74	Play Musical Instruments	30	0.48	50	0.24	51	0.30	29	0.29	18	0.18	62	0.36
75	Remember Important Dates	15	0.24	64	0.31	57	0.33	22	0.22	32	0.33	47	0.27
76	Creative Activities	17	0.27	61	0.29	41	0.24	38	0.38	21	0.21	58	0.34
77	Dressing Style	7	0.11	72	0.35	61	0.36	18	0.18	29	0.30	50	0.29
78	Make People Laugh	20	0.32	55	0.26	65	0.38	11	0.11	23	0.23	53	0.31

79	Laughter And Fun	9	0.14	65	0.31	64	0.37	12	0.12	32	0.33	44	0.25
80	Golf	49	0.79	26	0.12	24	0.14	51	0.51	36	0.37	39	0.23
81	Gym	22	0.35	51	0.24	49	0.29	24	0.24	31	0.32	42	0.24
82	Empathy	9	0.14	60	0.29	42	0.25	27	0.27	22	0.22	47	0.27
83	Contribute To Family	18	0.29	49	0.24	37	0.22	30	0.30	22	0.22	45	0.26
84	Remember Where Put Things	15	0.24	51	0.24	44	0.26	22	0.22	22	0.22	44	0.25
85	Follow A Conversation	9	0.14	55	0.26	52	0.30	13	0.13	21	0.21	44	0.25
86	Artwork	16	0.26	46	0.22	32	0.19	30	0.30	20	0.20	42	0.24
87	Mountain Sports	17	0.27	43	0.21	51	0.30	9	0.09	19	0.19	41	0.24
88	Dancing	5	0.08	52	0.25	36	0.21	22	0.22	17	0.17	41	0.24
89	Kind And Caring	13	0.21	44	0.21	39	0.23	18	0.18	16	0.16	41	0.24
90	Coffee With Friends	9	0.14	46	0.22	35	0.20	20	0.20	18	0.18	37	0.21
91	Follow Tv And News	17	0.27	37	0.18	34	0.20	20	0.20	14	0.14	40	0.23
92	Getting Dressed	7	0.11	47	0.23	44	0.26	10	0.10	21	0.21	33	0.19
93	Remember Day To Day Things	7	0.11	46	0.22	33	0.19	20	0.20	25	0.25	28	0.16
94	Yoga	2	0.03	51	0.24	33	0.19	20	0.20	20	0.20	33	0.19
95	Use Public Transport	11	0.18	36	0.17	31	0.18	18	0.18	13	0.13	36	0.21
96	Take Care Of Things	5	0.08	44	0.21	34	0.20	15	0.15	17	0.17	32	0.19
97	Mixboard Games And Cards	19	0.31	30	0.14	12	0.07	37	0.37	13	0.13	36	0.21
98	Express Opinions	12	0.19	35	0.17	30	0.18	17	0.17	14	0.14	33	0.19
99	Role In The Community	16	0.26	30	0.14	20	0.12	26	0.26	11	0.11	35	0.20
100	Plan Holidays	7	0.11	37	0.18	26	0.15	18	0.18	16	0.16	28	0.16
101	Same Person	11	0.18	31	0.15	31	0.18	11	0.11	10	0.10	32	0.19
102	Craftwork	10	0.16	31	0.15	19	0.11	22	0.22	18	0.18	23	0.13
103	Mixcookingbaking	6	0.10	34	0.16	26	0.15	14	0.14	13	0.13	27	0.16
104	Plan Family Life	10	0.16	30	0.14	30	0.18	10	0.10	20	0.20	20	0.12
105	Intelligence	16	0.26	23	0.11	24	0.14	15	0.15	11	0.11	28	0.16
106	Contribute To Conversation	7	0.11	31	0.15	24	0.14	14	0.14	10	0.10	28	0.16
107	Remember Recent Events	6	0.10	31	0.15	26	0.15	11	0.11	14	0.14	23	0.13
108	Remember Names	9	0.14	27	0.13	19	0.11	17	0.17	15	0.15	21	0.12
109	Prioritise And Analyse Tasks	7	0.11	28	0.13	26	0.15	9	0.09	18	0.18	17	0.10
110	Debate Politics	13	0.21	22	0.11	21	0.12	14	0.14	9	0.09	26	0.15
111	Happy And Loved	9	0.14	25	0.12	28	0.16	6	0.06	12	0.12	22	0.13
112	Not Be A Burden	12	0.19	21	0.10	19	0.11	14	0.14	15	0.15	18	0.10
113	Mixgardeningother	10	0.16	22	0.11	15	0.09	17	0.17	11	0.11	21	0.12
114	Share Memories	4	0.06	28	0.13	24	0.14	8	0.08	8	0.08	24	0.14
115	Remember Books	9	0.14	23	0.11	21	0.12	11	0.11	17	0.17	15	0.09
116	Make Tea	2	0.03	29	0.14	26	0.15	6	0.06	13	0.13	19	0.11
117	Remember Love	8	0.13	24	0.12	24	0.14	8	0.08	21	0.21	11	0.06
118	Understand Tv Programmes	7	0.11	25	0.12	15	0.09	17	0.17	14	0.14	18	0.10
119	Concentrate And Understand Books	18	0.29	14	0.07	16	0.09	16	0.16	13	0.13	19	0.11
120	Academic Activities	11	0.18	21	0.10	23	0.13	9	0.09	9	0.09	23	0.13
121	Maintain Contact	4	0.06	27	0.13	15	0.09	16	0.16	14	0.14	17	0.10
122	Situational Awareness	6	0.10	24	0.12	23	0.13	7	0.07	12	0.12	18	0.10
123	Control Budgets	5	0.08	24	0.12	19	0.11	10	0.10	17	0.17	12	0.07
124	Plan Leisure Time	10	0.16	17	0.08	16	0.09	11	0.11	11	0.11	16	0.09

125	Assess Complex Issues	13	0.21	14	0.07	18	0.11	9	0.09	9	0.09	18	0.10
126	Mixgalleriesmuseums	4	0.06	21	0.10	17	0.10	9	0.09	4	0.04	22	0.13
127	Organise Home	6	0.10	19	0.09	18	0.11	7	0.07	11	0.11	14	0.08
128	Exercise Classes	2	0.03	23	0.11	16	0.09	9	0.09	8	0.08	17	0.10
129	Sport Watching	14	0.23	10	0.05	20	0.12	4	0.04	13	0.13	11	0.06
130	Run Household	5	0.08	18	0.09	14	0.08	9	0.09	12	0.12	11	0.06
131	Group Activities	6	0.10	17	0.08	11	0.06	12	0.12	9	0.09	14	0.08
132	Mixconcertstheatrecinemamuseums	5	0.08	17	0.08	9	0.05	13	0.13	4	0.04	18	0.10
133	Motherhood	0	0.00	22	0.11	17	0.10	5	0.05	10	0.10	12	0.07
134	Wit And Humour	7	0.11	14	0.07	11	0.06	10	0.10	5	0.05	16	0.09
135	Gaming	10	0.16	11	0.05	16	0.09	5	0.05	8	0.08	13	0.08
136	Academic Activites	11	0.18	8	0.04	6	0.04	13	0.13	6	0.06	13	0.08
137	Mix Love Other	2	0.03	16	0.08	14	0.08	4	0.04	6	0.06	12	0.07
138	Baking	1	0.02	17	0.08	17	0.10	1	0.01	7	0.07	11	0.06
139	Retain A Good Memory	7	0.11	11	0.05	9	0.05	9	0.09	9	0.09	9	0.05
140	Remembering Words	5	0.08	12	0.06	11	0.06	6	0.06	2	0.02	15	0.09
141	Woman'S Role In Family	2	0.03	15	0.07	13	0.08	4	0.04	6	0.06	11	0.06
142	Pub	8	0.13	9	0.04	11	0.06	6	0.06	7	0.07	10	0.06
143	Racket Sports	5	0.08	11	0.05	11	0.06	5	0.05	8	0.08	8	0.05
144	Remain Positive	0	0.00	16	0.08	12	0.07	4	0.04	1	0.01	15	0.09
145	Dancing	2	0.03	13	0.06	4	0.02	12	0.12	5	0.05	11	0.06
146	Remember Conversations	4	0.06	11	0.05	9	0.05	6	0.06	6	0.06	9	0.05
147	In Control Of Life	6	0.10	9	0.04	9	0.05	6	0.06	4	0.04	11	0.06
148	Water Sports	11	0.18	4	0.02	12	0.07	3	0.03	8	0.08	7	0.04
149	Follow Instructions	4	0.06	10	0.05	10	0.06	4	0.04	6	0.06	8	0.05
150	Mixconcertsfestivals	3	0.05	11	0.05	8	0.05	6	0.06	4	0.04	10	0.06
151	Discuss Literature And Science	4	0.06	10	0.05	9	0.05	5	0.05	4	0.04	10	0.06
152	Remember Routes	5	0.08	9	0.04	6	0.04	8	0.08	5	0.05	9	0.05
153	Sex Life	8	0.13	5	0.02	6	0.04	7	0.07	5	0.05	8	0.05
154	Mixyogapilates	1	0.02	12	0.06	4	0.02	9	0.09	4	0.04	9	0.05
155	Remember Peoples Name And Face	2	0.03	11	0.05	8	0.05	5	0.05	4	0.04	9	0.05
156	Plan Meals	3	0.05	10	0.05	7	0.04	6	0.06	4	0.04	9	0.05
157	Staying Safe	1	0.02	10	0.05	11	0.06	1	0.01	2	0.02	10	0.06
158	New Friends	3	0.05	8	0.04	8	0.05	3	0.03	2	0.02	9	0.05
159	Bowls	6	0.10	5	0.02	2	0.01	9	0.09	6	0.06	5	0.03
160	Speak Foreign Languages	2	0.03	9	0.04	6	0.04	5	0.05	5	0.05	6	0.03
161	Fishing	11	0.18	0	0.00	6	0.04	5	0.05	8	0.08	3	0.02
162	Read Music	4	0.06	7	0.03	8	0.05	3	0.03	4	0.04	7	0.04
163	Good Company	4	0.06	7	0.03	9	0.05	2	0.02	3	0.03	8	0.05
164	Pilates	1	0.02	9	0.04	5	0.03	5	0.05	2	0.02	8	0.05
165	Contribute To Society	3	0.05	7	0.03	8	0.05	2	0.02	1	0.01	9	0.05
166	Listen To Radio	2	0.03	8	0.04	6	0.04	4	0.04	3	0.03	7	0.04
167	Mixridinghorseridingbike	4	0.06	5	0.02	7	0.04	2	0.02	7	0.07	2	0.01
168	Tai Chi	1	0.02	7	0.03	4	0.02	4	0.04	1	0.01	7	0.04
169	Manage Appointments	1	0.02	7	0.03	5	0.03	3	0.03	3	0.03	5	0.03
170	Manage Diary	1	0.02	6	0.03	5	0.03	2	0.02	2	0.02	5	0.03

171	Diy	5	0.08	1	0.00	1	0.01	5	0.05	4	0.04	2	0.01
172	Mixcinematheatre	1	0.02	5	0.02	2	0.01	4	0.04	2	0.02	4	0.02
173	Retain Maths Ability	1	0.02	5	0.02	4	0.02	2	0.02	0	0.00	6	0.03
174	Photography	2	0.03	3	0.01	4	0.02	1	0.01	1	0.01	4	0.02
175	Follow Needlework Pattern	0	0.00	5	0.02	1	0.01	4	0.04	2	0.02	3	0.02
176	Entertain	2	0.03	2	0.01	1	0.01	3	0.03	2	0.02	2	0.01
177	Follow A Recipe	0	0.00	4	0.02	3	0.02	1	0.01	1	0.01	3	0.02
178	Winter Sports	0	0.00	3	0.01	2	0.01	1	0.01	2	0.02	1	0.01
179	Hearing	3	0.05	0	0.00	1	0.01	2	0.02	2	0.02	1	0.01
180	Mental Arithmetic	1	0.02	1	0.00	1	0.01	1	0.01	1	0.01	1	0.01
181	Camping	1	0.02	0	0.00	1	0.01	0	0.00	1	0.01	0	0.00
182	Compassionate	1	0.02	0	0.00	1	0.01	0	0.00	0	0.00	1	0.01
183	Play Games	1	0.02	0	0.00	1	0.01	0	0.00	0	0.00	1	0.01
184	Not Elsewhere Categorised	559	9.00	1572	7.55	1220	7.13	916	9.17	761	7.75	1375	7.96
	Total	6213	100.00	20829	100.00	17120	100.00	9985	100.00	9825	100.00	17280	100.00
