# Supplementary material for: A UK-Wide Study Employing Natural Language Processing to Determine What Matters to People about Brain Health to Improve Drug Development: The Electronic Person-Specific Outcome Measure (ePSOM) Programme
Source: J Prev Alzheimers Dis. 2021 Jun 9;8(4):448–56. doi: 10.14283/jpad.2021.30 (PMC12280764; doi:10.14283/jpad.2021.30)
Supplement: Supplementary file 4 — Statistically significant differences in identifying themes as particularly important, comparing responses from demographic group dyads [file mmc4.pdf]

Statistically significant differences in identifying themes as particularly important, comparing responses from demographic group dyads

Theme	Male %	Female %	Gender p	Age <= 64 %	Age >= 65 %	Age p	No Degree %	Degree %	Educ. p
Family connection	4.506680	4.743387		5.058411	4.076114		5.251908	4.380787	< 0.01
Driving	5.311444	4.493735	< 0.01	4.129673	5.598398	< 0.01	5.781170	4.039352	< 0.01
Socialising	3.959440	4.056844		4.053738	3.985979		4.600509	3.703704	< 0.01
Reading	3.637534	4.080849		3.843458	4.196294		3.501272	4.241898	< 0.01
Friendships	3.042009	3.860003	< 0.01	3.539720	3.885829	< 0.01	3.552163	3.732639	
Walking	3.396105	2.880599		2.949766	3.094642		2.972010	3.020833	
Maintain independence	1.915339	3.082241	< 0.01	2.978972	2.523786		2.819338	2.806713	
Mix family connections friendships	2.333816	2.616544		2.762850	2.173260	< 0.01	2.687023	2.465278	
Conversation and chat	2.076292	2.424504		2.511682	2.073110		2.361323	2.343750	
Follow a storyline	1.979720	1.925200		2.032710	1.772659	< 0.01	1.720102	2.060185	
Personal hygiene	1.062289	2.098036	< 0.01	2.172897	1.311968	< 0.01	1.679389	1.956019	
Cooking	1.400290	1.872389		1.997664	1.362043	< 0.01	1.455471	1.938657	< 0.01
Use technology	1.786577	1.646743		1.442757	2.073110	< 0.01	1.547074	1.747685	
Recognise people	1.609528	1.603534		1.828271	1.211818	< 0.01	1.628499	1.585648	
Make decisions	1.062289	1.675549	< 0.01	1.740654	1.171758	< 0.01	1.353690	1.631944	
Meaningful conversations	1.673910	1.430698		1.623832	1.241863		1.526718	1.458333	
Feel wanted and needed	1.593433	1.421096		1.489486	1.412118		1.292621	1.556713	
Gardening	1.046193	1.469106		1.074766	1.902854	< 0.01	1.394402	1.371528	
Manage finances	1.416385	1.320275		1.133178	1.692539	< 0.01	1.363868	1.325231	
Communicate effectively	1.432480	1.152240		1.028037	1.532298	< 0.01	1.241730	1.197917	
Live at home	0.853050	1.224255		1.086449	1.231848		0.997455	1.221065	
Grandchildren	1.014003	1.065822		0.876168	1.352028	< 0.01	1.170483	0.983796	
Exercise	1.078384	1.027414		1.139019	0.861292		0.956743	1.082176	
Walk dogs	0.676002	1.099429	< 0.01	1.191589	0.681022	< 0.01	1.312977	0.827546	< 0.01
Volunteering	0.997908	0.955399		0.794393	1.251878	< 0.01	0.743003	1.087963	< 0.01
Personal attributes and social skills	0.933526	0.965001		0.969626	0.931397		0.966921	0.949074	
Remember peoples name	1.174956	0.796966	< 0.01	0.811916	1.001502		0.966921	0.833333	
Remember past	0.949622	0.854578		0.952103	0.751127		0.844784	0.896991	
Support family	0.611621	0.955399		0.957944	0.741112		0.854962	0.891204	
Music	1.287623	0.720150	< 0.01	0.823598	0.901352		0.631043	0.978009	< 0.01
Travelling	0.885241	0.830573		0.806075	0.921382		0.722646	0.920139	
Use toilet	0.531144	0.844976		0.870327	0.610916		0.641221	0.850694	
Sense of humour	0.643811	0.729752		0.841121	0.500751	< 0.01	0.773537	0.682870	
Pets	0.466763	0.777762		0.887850	0.390586	< 0.01	0.508906	0.815972	< 0.01
Shopping	0.338001	0.806568	< 0.01	0.741822	0.620931	< 0.01	0.926209	0.567130	< 0.01

Working	0.611621	0.720150		1.028037	0.130195	< 0.01	0.753181	0.665509	
Help others	0.772574	0.672140		0.677570	0.721082		0.865140	0.596065	
Cognitive games	0.901336	0.628931		0.449766	1.101652	< 0.01	0.783715	0.636574	
Hobbies	0.627716	0.686543		0.601636	0.791187		0.681934	0.665509	
Dining	0.515049	0.686543		0.712617	0.540811	< 0.01	0.641221	0.653935	
Give advice	0.949622	0.528110	< 0.01	0.625000	0.630946		0.834606	0.509259	< 0.01
Caring responsibilities	0.611621	0.633732		0.490654	0.861292	< 0.01	0.773537	0.543981	
Household chores	0.386287	0.667339		0.613318	0.590886		0.508906	0.659722	
Analyse and solve problems	0.595526	0.585722		0.595794	0.580871		0.600509	0.584491	
Maintain dignity	0.627716	0.556916		0.554907	0.600901		0.569975	0.572917	
Going on holidays	0.515049	0.576120		0.566589	0.550826		0.763359	0.445602	< 0.01
Spouse	0.579430	0.552115		0.630841	0.430646		0.458015	0.613426	
Staying active	0.836955	0.432090	< 0.01	0.455607	0.640961		0.468193	0.555556	
Religious participation	0.338001	0.571319		0.461449	0.610916	< 0.01	0.427481	0.567130	
Look good	0.354096	0.556916		0.455607	0.600901		0.631043	0.439815	
Cycling	0.933526	0.379279	< 0.01	0.595794	0.350526	< 0.01	0.508906	0.503472	
Understand current affairs	0.756478	0.403284	< 0.01	0.502336	0.450676		0.417303	0.520833	
Rational thinking	0.756478	0.398483	< 0.01	0.502336	0.440661		0.468193	0.486111	
Needlework	0.032191	0.576120	< 0.01	0.467290	0.430646		0.610687	0.364583	< 0.01
Planning and organising skills	0.321906	0.446493		0.408879	0.430646		0.376590	0.439815	
Good listener	0.321906	0.436891		0.443925	0.360541		0.346056	0.451389	
Singing	0.257525	0.460896		0.327103	0.560841	< 0.01	0.356234	0.445602	
Feel valued and self-worth	0.386287	0.398483		0.420561	0.360541		0.346056	0.428241	
Leisure travel	0.418477	0.388881		0.321262	0.520781		0.386768	0.399306	
Plan future	0.354096	0.369677		0.426402	0.260391	< 0.01	0.295165	0.405093	
Confidence	0.321906	0.355274		0.321262	0.390586		0.264631	0.393519	
Watch tv	0.498954	0.292861		0.303738	0.410616		0.305344	0.364583	
Sports	0.788669	0.211244	< 0.01	0.286215	0.440661		0.488550	0.260417	< 0.01
Mental agility	0.515049	0.288060		0.344626	0.340511		0.366412	0.329861	
Running	0.515049	0.288060		0.449766	0.150225	< 0.01	0.305344	0.358796	
Take part in activities	0.321906	0.340871		0.309579	0.390586	< 0.01	0.305344	0.358796	
Traveling	0.370192	0.326468		0.356308	0.300451		0.173028	0.428241	< 0.01
Capacity to understand text	0.338001	0.326468		0.315421	0.350526		0.254453	0.370370	
Swimming	0.257525	0.336070		0.373832	0.250376	< 0.01	0.386768	0.295139	
Ability to learn new skills	0.386287	0.297662		0.315421	0.320481		0.254453	0.353009	
Mixtheatrecinema	0.193143	0.340871		0.303738	0.320481		0.274809	0.329861	
Writing	0.386287	0.283259		0.280374	0.350526		0.213740	0.358796	
Analytical skills	0.450668	0.254453		0.268692	0.350526		0.244275	0.329861	
Play musical instruments	0.482859	0.240050	< 0.01	0.297897	0.290436		0.183206	0.358796	
Remember important dates	0.241429	0.307264		0.332944	0.220330	< 0.01	0.325700	0.271991	
Creative activities	0.273620	0.292861		0.239486	0.380571	< 0.01	0.213740	0.335648	
Dressing style	0.112667	0.345672	< 0.01	0.356308	0.180270	< 0.01	0.295165	0.289352	
Make people laugh	0.321906	0.264055		0.379673	0.110165	< 0.01	0.234097	0.306713	
Laughter and fun	0.144858	0.312065		0.373832	0.120180	< 0.01	0.325700	0.254630	
Golf	0.788669	0.124826	< 0.01	0.140187	0.510766	< 0.01	0.366412	0.225694	
Gym	0.354096	0.244851		0.286215	0.240361	< 0.01	0.315522	0.243056	

Empathy	0.144858	0.288060		0.245327	0.270406		0.223919	0.271991	
Contribute to family	0.289715	0.235249		0.216121	0.300451	< 0.01	0.223919	0.260417	
Remember where put things	0.241429	0.244851		0.257009	0.220330		0.223919	0.254630	
Follow a conversation	0.144858	0.264055		0.303738	0.130195		0.213740	0.254630	
Artwork	0.257525	0.220846		0.186916	0.300451		0.203562	0.243056	
Mountain sports	0.273620	0.206443		0.297897	0.090135	< 0.01	0.193384	0.237269	
Dancing	0.080476	0.249652		0.210280	0.220330		0.173028	0.237269	
Kind and caring	0.209239	0.211244		0.227804	0.180270		0.162850	0.237269	
Coffee with friends	0.144858	0.220846		0.204439	0.200300		0.183206	0.214120	
Follow tv and news	0.273620	0.177637		0.198598	0.200300		0.142494	0.231481	
Getting dressed	0.112667	0.225647		0.257009	0.100150	< 0.01	0.213740	0.190972	
Remember day to day things	0.112667	0.220846		0.192757	0.200300		0.254453	0.162037	
Yoga	0.032191	0.244851	< 0.01	0.192757	0.200300		0.203562	0.190972	
Use public transport	0.177048	0.172836		0.181075	0.180270		0.132316	0.208333	
Take care of things	0.080476	0.211244		0.198598	0.150225		0.173028	0.185185	
Mixboard games and cards	0.305810	0.144030		0.070093	0.370556	< 0.01	0.132316	0.208333	
Express opinions	0.193143	0.168035		0.175234	0.170255		0.142494	0.190972	
Role in the community	0.257525	0.144030		0.116822	0.260391	< 0.01	0.111959	0.202546	
Plan holidays	0.112667	0.177637		0.151869	0.180270		0.162850	0.162037	
Same person	0.177048	0.148831		0.181075	0.110165		0.101781	0.185185	
Craftwork	0.160953	0.148831		0.110981	0.220330		0.183206	0.133102	
Mixcookingbaking	0.096572	0.163234		0.151869	0.140210		0.132316	0.156250	
Plan family life	0.160953	0.144030		0.175234	0.100150		0.203562	0.115741	
Intelligence	0.257525	0.110423		0.140187	0.150225		0.111959	0.162037	
Contribute to conversation	0.112667	0.148831		0.140187	0.140210		0.101781	0.162037	
Remember recent events	0.096572	0.148831		0.151869	0.110165		0.142494	0.133102	
Remember names	0.144858	0.129627		0.110981	0.170255	< 0.01	0.152672	0.121528	
Prioritise and analyse tasks	0.112667	0.134428		0.151869	0.090135		0.183206	0.098380	
Debate politics	0.209239	0.105622		0.122664	0.140210	< 0.01	0.091603	0.150463	
Happy and loved	0.144858	0.120025		0.163551	0.060090		0.122137	0.127315	
Not be a burden	0.193143	0.100821		0.110981	0.140210		0.152672	0.104167	
Mixgardeningother	0.160953	0.105622		0.087617	0.170255		0.111959	0.121528	
Share memories	0.064381	0.134428		0.140187	0.080120		0.081425	0.138889	
Remember books	0.144858	0.110423		0.122664	0.110165		0.173028	0.086806	
Make tea	0.032191	0.139229		0.151869	0.060090	< 0.01	0.132316	0.109954	
Remember love	0.128762	0.115224		0.140187	0.080120		0.213740	0.063657	< 0.01
Understand tv programmes	0.112667	0.120025		0.087617	0.170255		0.142494	0.104167	
Concentrate and understand books	0.289715	0.067214	< 0.01	0.093458	0.160240		0.132316	0.109954	
Academic activities	0.177048	0.100821		0.134346	0.090135		0.091603	0.133102	
Maintain contact	0.064381	0.129627		0.087617	0.160240		0.142494	0.098380	
Situational awareness	0.096572	0.115224		0.134346	0.070105		0.122137	0.104167	
Control budgets	0.080476	0.115224		0.110981	0.100150		0.173028	0.069444	
Plan leisure time	0.160953	0.081617		0.093458	0.110165		0.111959	0.092593	
Assess complex issues	0.209239	0.067214	< 0.01	0.105140	0.090135		0.091603	0.104167	
Mixgalleriesmuseums	0.064381	0.100821		0.099299	0.090135		0.040712	0.127315	
Organise home	0.096572	0.091219		0.105140	0.070105		0.111959	0.081019	

Exercise classes	0.032191	0.110423		0.093458	0.090135		0.081425	0.098380	
Sport watching	0.225334	0.048010	< 0.01	0.116822	0.040060	< 0.01	0.132316	0.063657	
Run household	0.080476	0.086418		0.081776	0.090135		0.122137	0.063657	
Group activities	0.096572	0.081617		0.064252	0.120180	< 0.01	0.091603	0.081019	
Mixconcertstheatrecinemamuseums	0.080476	0.081617		0.052570	0.130195	< 0.01	0.040712	0.104167	
Motherhood	0.000000	0.105622		0.099299	0.050075		0.101781	0.069444	
Wit and humour	0.112667	0.067214		0.064252	0.100150		0.050891	0.092593	
Gaming	0.160953	0.052811		0.093458	0.050075		0.081425	0.075231	
Academic activites	0.177048	0.038408	< 0.01	0.035047	0.130195		0.061069	0.075231	
Mix love other	0.032191	0.076816		0.081776	0.040060		0.061069	0.069444	
Baking	0.016095	0.081617		0.099299	0.010015	< 0.01	0.071247	0.063657	
Retain a good memory	0.112667	0.052811		0.052570	0.090135		0.091603	0.052083	
Remembering words	0.080476	0.057612		0.064252	0.060090		0.020356	0.086806	
Woman's role in family	0.032191	0.072015		0.075935	0.040060		0.061069	0.063657	
Pub	0.128762	0.043209		0.064252	0.060090		0.071247	0.057870	
Racket sports	0.080476	0.052811		0.064252	0.050075		0.081425	0.046296	
Remain positive	0.000000	0.076816		0.070093	0.040060		0.010178	0.086806	
Dancing	0.032191	0.062413		0.023364	0.120180		0.050891	0.063657	
Remember conversations	0.064381	0.052811		0.052570	0.060090		0.061069	0.052083	
In control of life	0.096572	0.043209		0.052570	0.060090		0.040712	0.063657	
Water sports	0.177048	0.019204	< 0.01	0.070093	0.030045		0.081425	0.040509	
Follow instructions	0.064381	0.048010		0.058411	0.040060		0.061069	0.046296	
Mixconcertsfestivals	0.048286	0.052811		0.046729	0.060090		0.040712	0.057870	
Discuss literature and science	0.064381	0.048010		0.052570	0.050075		0.040712	0.057870	
Remember routes	0.080476	0.043209		0.035047	0.080120		0.050891	0.052083	
Sex life	0.128762	0.024005	< 0.01	0.035047	0.070105		0.050891	0.046296	
Mixyogapilates	0.016095	0.057612		0.023364	0.090135		0.040712	0.052083	
Remember peoples name and face	0.032191	0.052811		0.046729	0.050075		0.040712	0.052083	
Plan meals	0.048286	0.048010		0.040888	0.060090		0.040712	0.052083	
Staying safe	0.016095	0.048010		0.064252	0.010015		0.020356	0.057870	
New friends	0.048286	0.038408		0.046729	0.030045		0.020356	0.052083	
Bowls	0.096572	0.024005		0.011682	0.090135	< 0.01	0.061069	0.028935	
Speak foreign languages	0.032191	0.043209		0.035047	0.050075		0.050891	0.034722	
Fishing	0.177048	0.000000	< 0.01	0.035047	0.050075		0.081425	0.017361	
Read music	0.064381	0.033607		0.046729	0.030045		0.040712	0.040509	
Good company	0.064381	0.033607		0.052570	0.020030		0.030534	0.046296	
Pilates	0.016095	0.043209		0.029206	0.050075		0.020356	0.046296	
Contribute to society	0.048286	0.033607		0.046729	0.020030		0.010178	0.052083	
Listen to radio	0.032191	0.038408		0.035047	0.040060		0.030534	0.040509	
Mixridinghorseridingbike	0.064381	0.024005		0.040888	0.020030		0.071247	0.011574	
Tai chi	0.016095	0.033607		0.023364	0.040060		0.010178	0.040509	
Manage appointments	0.016095	0.033607		0.029206	0.030045		0.030534	0.028935	
Manage diary	0.016095	0.028806		0.029206	0.020030		0.020356	0.028935	
Diy	0.080476	0.004801	< 0.01	0.005841	0.050075		0.040712	0.011574	
Mixcinematheatre	0.016095	0.024005		0.011682	0.040060		0.020356	0.023148	
Retain maths ability	0.016095	0.024005		0.023364	0.020030		0.000000	0.034722	

Photography	0.032191	0.014403		0.023364	0.010015		0.010178	0.023148	
Follow needlework pattern	0.000000	0.024005		0.005841	0.040060		0.020356	0.017361	
Entertain	0.032191	0.009602		0.005841	0.030045	< 0.01	0.020356	0.011574	
Follow a recipe	0.000000	0.019204		0.017523	0.010015		0.010178	0.017361	
Winter sports	0.000000	0.014403		0.011682	0.010015		0.020356	0.005787	
Hearing	0.048286	0.000000		0.005841	0.020030		0.020356	0.005787	
Mental arithmetic	0.016095	0.004801		0.005841	0.010015		0.010178	0.005787	
Camping	0.016095	0.000000		0.005841	0.000000		0.010178	0.000000	
Compassionate	0.016095	0.000000		0.005841	0.000000		0.000000	0.005787	
Play games	0.016095	0.000000		0.005841	0.000000		0.000000	0.005787	
